# Subclinical Detection of Hydroxychloroquine-Induced Retinopathy in Patients with Systemic Lupus Erythematous Using Multifocal Electroretinography and Optical Coherence Tomography

**DOI:** 10.3390/jcm13247663

**Published:** 2024-12-16

**Authors:** Suk Hoon Jung, Young-Hoon Park, Young Gun Park

**Affiliations:** 1 Department of Ophthalmology and Visual Science, Seoul St. Mary’s Hospital, College of Medicine, The Catholic University of Korea, Seoul 06591, Republic of Korea; 2 Catholic Institute for Visual Science, College of Medicine, The Catholic University of Korea, Seoul 06591, Republic of Korea

**Keywords:** hydroxychloroquine, systemic lupus erythematosus, multifocal electroretinogram

## Abstract

**Background/Objectives:** Although hydroxychloroquine (HCQ) is used to treat systemic lupus erythematosus (SLE), it is associated with retinal toxicity. Early diagnosis can prevent the further progression of HCQ-associated retinopathy by discontinuing HCQ treatments. This study aimed to evaluate the early diagnostic parameters in patients with SLE treated with HCQ and identify the best approach using multifocal electroretinography (mfERG) and swept-source optical coherence tomography (SS-OCT) to reflect subclinical retinal toxicity. **Methods:** Thirty-eight patients with SLE (76 eyes) and 18 healthy controls (36 eyes) were enrolled. They were referred for HCQ retinopathy screening without visual field defects. The patients were tested using a standard 61-hexagon mfERG stimulus and SS-OCT. Ten groups of the mfERG responses from the sectors were averaged to compare the quadrants, hemiretinal areas, consecutive ring amplitudes, and ring ratios (R1/R2–R5) from the center to the periphery. Additionally, the ganglion cell complex (GCC) analyses were performed using SS-OCT. **Results:** No difference was observed in GCC thickness on the OCT images, in the P1 amplitudes, and in the implicit time of mfERG. However, the R1/Rx ring ratios, except the R1/R2 ratio, showed significant differences among the three groups (*p* = 0.759, 0.018, 0.029, and 0.029, respectively). The R1/R3, R1/R4, and R1/R5 ring ratios demonstrated a correlation with the duration of HCQ therapy (r = −0.303, −0.279, and −0.266; *p* = 0.003, 0.006, and 0.009). The areas under the receiver operating characteristic curve of the ring ratios R1/R3–R5 were 0.730, 0.702, and 0.724, respectively (*p* = 0.004, 0.012, and 0.006), indicating the likelihood of being categorized as a high-risk group for subclinical HCQ retinopathy. **Conclusions:** The ring ratio of mfERG reflects the subclinical electrophysiological alterations induced by HCQ and can become more clinically useful by simplifying screening examinations.

## 1. Introduction

Hydroxychloroquine (HCQ) is one of the disease-modifying anti-rheumatic drugs that is used to treat various rheumatological diseases, including systemic lupus erythematosus (SLE) and rheumatoid arthritis [1]. Despite its therapeutic benefits, physicians must carefully consider the risks associated with HCQ therapy. An increased risk of retinopathy resulted in updated ophthalmology guidelines that recommended a maximum daily dose of 5.0 mg/kg of actual body weight for HCQ treatments. Although HCQ has a lower risk of toxicity than chloroquine, it can sometimes cause damage to the outer retina, also known as HCQ retinopathy [2,3].

Retinal toxicity is an important side effect of HCQ; the risk ratio increases according to the dose and duration of the therapy [2], ranging from 1% to 7.5% in patients with long-term exposure [4]. However, whether a true safe dose for the long-term use of HCQ exists remains unclear. The risk factors for the incidence of retinal toxicity include HCQ treatment for >5 years, an excessive daily dose, concurrent tamoxifen use, and pre-existing retinal and other systemic diseases.

Most patients in the early stages of retinal toxicity have negligible visual symptoms, making a diagnosis difficult. Additionally, HCQ retinal toxicity can progress despite drug-use discontinuation, and the severity of the structural or functional loss depends on the timing at which the disease is noticed [5,6]. Moreover, it is a serious ophthalmological concern as it is untreatable. Nonetheless, central vision can be preserved if the damage is recognized before changes occur in the retinal pigment epithelium (RPE) [5]. Therefore, proper screening, the early detection of retinal toxicity, and the termination of HCQ therapy before structural retinal damage occurs are crucial to prevent disease progression.

Previous histopathological studies of human retinas with chloroquine retinopathy have shown the destruction of photoreceptors and neuroretina [7,8]. Rosenthal et al. reported that chloroquine caused an initial dramatic effect on the retinal ganglion cells (RGCs), with a subsequent degeneration of the RGCs and photoreceptors in rhesus monkeys [9]. Therefore, the current 2016 American Academy of Ophthalmology (AAO) guidelines recommend that a baseline examination of fundus status should be performed within the first year of initiating therapy using automated visual field (VF) and spectral domain optical coherence tomography (SD-OCT). In the absence of additional risk factors, annual screenings can begin after 5 years of exposure with proper VF according to an SD-OCT. Although SD-OCT can accurately detect retinal toxicity based on structural changes, it is not as sensitive as functional tests, such as VF and multifocal electroretinography (mfERG) [10].

Nonetheless, assessing retinal structures has become easier with the introduction of SD-OCT devices [11,12]. Changes in the outer retina, including the loss of the outer cone segment and the ellipsoid zone, which progress to parafoveal thinning of the outer nuclear layer and the consequent RPE damage, are the earliest detectable signs. In contrast, animal studies have shown that HCQ toxicity initially affects the inner retinal layers, such as the ganglion cell layer (GCL), and later progresses to photoreceptor and RPE damage. These results suggest that early HCQ toxicity manifests as changes in the inner retinal layers in SD-OCT studies [13].

VF is easily available for screening; however, a patient’s subjective responses can result in obvious variations. In response to these limitations, mfERG is recommended to objectively confirm suspected field loss. Despite the sensitivity of mfERG, its widespread use is limited by difficulties in interpreting the results. Although several researchers have attempted to identify the appropriate early diagnostic parameters, they could not detect simple or subclinical changes in HCQ retinopathy. Moreover, the mfERG test was useful for detecting the early subtle electrophysiological changes secondary to retinal cell stress in patients undergoing HCQ therapy, which may also be an antecedent to outer retinal damage as represented by the changes in the RPE detectable on an OCT image.

Therefore, we aimed to evaluate a novel method for subclinical HCQ retinopathy detection using the mfERG findings that specifically targeted the parafoveal region according to the bull’s eye maculopathy screening test. Additionally, we focused on the structural changes in the inner retinal thickness, such as the changes to the GCL complex observed on an OCT image. These findings can simplify and expedite routine HCQ toxicity screening in patients with SLE.

## 2. Materials and Methods

### 2.1. Patients

This retrospective study included all patients referred to the Catholic University of Korea Eye Institute for HCQ retinopathy screening between August 2021 and March 2023. Patient data, including sex, age, best-corrected visual acuity (BCVA), HCQ duration (years), and history of systemic disease, were collected. All patients provided informed consent. The Institutional Research Ethics Board of the Catholic University of Korea approved this cross-sectional study protocol.

Patients from the rheumatology and ophthalmology departments who were screened for HCQ retinopathy and underwent imaging for HCQ toxicity with VF testing using static, automated threshold perimetry Humphrey Field Analyzer, Model 750I;Carl Zeiss Meditec, Dublin, CA, USA) and were confirmed to have no VF defects, such as paracentral scotoma, were included. Patients with ocular diseases, including cataracts, glaucoma, optic nerve and retinal diseases, and uveitis; with a prior history of ocular surgery; with high refractive errors (>±5 D sphere); with significant media opacities that interfered with OCT imaging; with a coexisting retinal disease precluding an appropriate evaluation of the retina; and with a prior history of retinal surgery were excluded from this study. Patients with a history of diabetes or tamoxifen administration were also excluded.

All participants underwent a comprehensive ophthalmologic examination, including a measurement of BCVA and dilated fundus photography. Subsequently, swept-source optical coherence tomography (SS-OCT) and mfERG measurements were performed. SS-OCT enabled more accurate 3D imaging due to a higher scan speed and greater sensitivity than SD-OCT. Since using HCQ for >5 years is a known risk factor for HCQ retinopathy, the 51 included patients (102 eyes) were categorized into the following three groups: group 1A, 19 patients with HCQ use <5 years (low risk); group 1B, 19 patients with HCQ use >5 years (high risk); and group 2, 13 healthy controls. Both eyes of all the participants were included in this study.

### 2.2. Thickness of Ganglion Cell Complex (GCC) Using Swept-Source OCT

SS-OCT (DRI OCT Triton, Topcon Inc., Tokyo, Japan) measurements were performed, and only high-quality images (signal strength ≥ 5) were included. This provides an overall map of the macular examinations, showing the macular and GCC analyses. A GCC analysis (nerve fiber layer [NFL] + GCL + inner plexiform layer [IPL]) (9 mm × 9 mm) is a thickness map of the inner retinal layer composed of the NFL, GCL, and IPL (inner limiting membrane to IPL/inner nuclear layer).

### 2.3. Multifocal Electroretinogram

The multifocal visual electrophysiology examination system (RETI-port/scan 21, Roland, Berlin, Germany) was used according to the International Society for Clinical Electrophysiology of Vision standard [14]. mfERG was conducted under photopic conditions, with the pupil of the study eye dilated and recorded using corneal contact lens electrodes. Stimulations were displayed at a luminance of 220 cd/m^2^. The studied eye was stimulated with 8 runs of 61 hexagonal elements covering 54° of the retina (a 27° radius from the fixation point), which increased with eccentricity. The mfERG outcome was the amplitude of the first-order kernel from the first to the fifth ring. The results were printed on a result sheet. The largest ring, ring 5 (R5), had an outer angle radius of 30°. Rings 4 to 1 had radii of 20° (R4), 9° (R3), 4.25° (R2), and 2.5° (R1), respectively.

**Figure 1:**
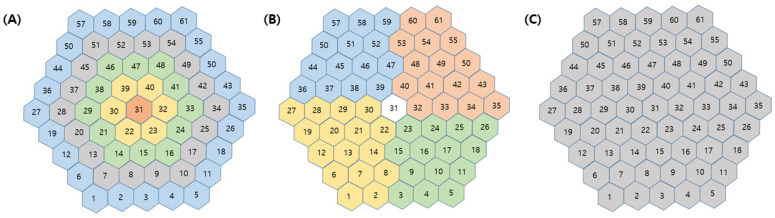
Definition of groups of multifocal electroretinography responses. (**A**) Five concentric rings from inner to outer (R1, R2, R3, R4, and R5), (**B**) four quadrants (superior temporal, inferior temporal, inferior nasal and superior nasal area), and (**C**) average in whole macular area.

Groups of mfERG responses from the sectors can be averaged to compare quadrants or successive rings from the center to the periphery. Accordingly, in our study, we considered the following 10 groups (Figure 1): (1) five concentric rings centered on the fovea for analysis from inner to outer: R1 (1 hexagon), R2 (6 hexagons, parafoveal ring), R3 (12 hexagons), R4 (18 hexagons), and R5 (24 hexagons); (2) four quadrants: superior temporal (ST), inferior temporal (IT), inferior nasal (IN), and superior nasal (SN); and (3) average (whole macular field). The groupings were obtained for each eye by averaging the mfERG sectors that comprised each grouping. The ring ratios were computed as the ratios of rings 1 through 4 to ring 5. The ring amplitudes (R1–R5) and ring ratios (R1/R2–5) were calculated automatically.

### 2.4. Statistical Analysis

Statistical analyses were performed using IBM SPSS Statistics for Windows, version 23.0 (IBM Corp., Armonk, NY, USA). Comparisons between groups were conducted using the independent sample *t*-test and an analysis of variance. Pearson’s correlation analysis was used to assess the relationships between the variables. The high-risk probability of developing subclinical retinal toxicity was predicted according to the area under the receiver operating characteristic (ROC) curve to analyze the ring ratio. Statistical significance was set at *p* < 0.05.

## 3. Results

### 3.1. Baseline Characteristics

Overall, 76 eyes (38 patients) were analyzed: 1 male and 37 female patients, with a mean age of 49.7 ± 14.4 (with a range of 23–85) years. Additionally, 18 healthy individuals (36 eyes) were included as the healthy controls. The enrolled patients were administered HCQ for SLE. The demographic and clinical characteristics of the participants are presented in Table 1.

### 3.2. Thickness of Macular GCC in SS-OCT

We measured the thickness in the ST, IN, inferior, and IT sectors to compare the macular GCC thickness parameters. Significant differences were not observed in the thickness of the parafoveal GCC layer between the patients and healthy controls. The average GCL thickness was relatively lower, but not significantly different, in those with HCQ-treated eyes than in the eyes of the healthy controls (Table 2).

### 3.3. Parameters of mfERG Metrics

Parameters for the P1 amplitudes and implicit time are presented in Table 3 and Table 4. The amplitudes in the ring and quadrant groups showed no significant differences. The average amplitudes also exhibited no differences among the groups.

In group 1A, the R1/Rx ring ratios were 2.67 ± 1.01, 4.58 ± 1.71, 6.06 ± 1.73, and 6.61 ± 1.90 for R2–R5, respectively; in group 1B, the corresponding values were 3.27 ± 3.98, 4.43 ± 3.41, 5.57 ± 2.03, and 6.1 ± 2.57. In group 2, the R1/Rx ring ratios were 2.69 ± 0.86, 4.74 ± 1.32, 6.71 ± 2.03, and 7.6 ± 3.21 for R2–R5, respectively. All the R1/Rx ratios, except the R1/R2 ratio, showed significant differences among the three groups (*p* = 0.759, 0.018, 0.029, and 0.029, respectively). Additionally, significant differences were observed between the two groups, groups 1A and B, based on the duration of HCQ treatment (<5 years or >5 years) (*p* = 0.023, 0.048, and 0.045); however, significant changes were not observed between groups 1A and 2. The R1/R3, R1/R4, and R1/R5 ring ratios of the new ring ratio demonstrated a correlation with the duration of HCQ therapy (r = −0.303, −0.279, and −0.266; *p* = 0.003, 0.006, and 0.009) (Table 5).

**Figure 2:**
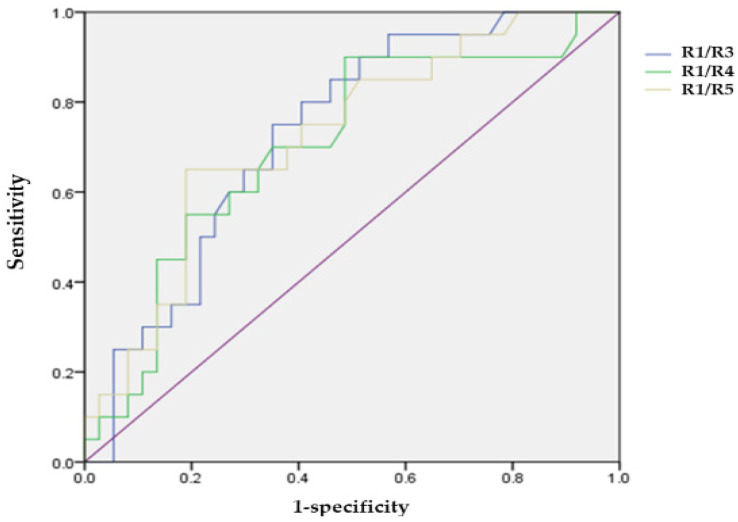
Receiver operating characteristic curve of ring ratios (R1/R3, R1/R4, and R1/R5) for the high-risk of hydroxychloroquine induced retinopathy.

In groups 1A and B, the areas under the ROC curve of the ring ratios R1/R3, R1/R4, and R1/R5 were 0.730, 0.702, and 0.724, respectively (*p* = 0.004, 0.012, and 0.006). Their cut-off values were 3.84 (R1/R3), 5.60 (R1/R4), and 6.09 (R1/R5), respectively, which showed a greater significance for the high risk of subclinical HCQ retinopathy (Figure 2).

## 4. Discussion

HCQ retinopathy, typically described as pericentral retinopathy, involves damages to the retina ≥ 8° from the center of the fovea. Previous findings emphasize the importance of the regular monitoring of patients undergoing long-term HCQ therapy to detect potential retinal changes in the early stages [4,15,16,17].

However, the exact mechanism underlying HCQ retinal toxicity remains unknown. HCQ is likely to bind to the melanin within the RPE and choroid such as melanin-rich ocular tissues [18]. The drug increases its concentration over time as a continuous drug reservoir until finally decompensating the retinal cell metabolism and destroying photoreceptors and the outer nuclear layer. In the chronic stages, atrophy of the RPE and neurosensory retina spreads outward over the whole retina. HCQ retinal atrophy is generally irreversible and may progress even after a discontinuation of the medication [2,19].

Previous studies have implicated damage to the outer retinal structures, such as the photoreceptors and RPE cells, whereas others have suspected damage to the RGCs, IPL, or retinal NFL [20,21]. Marmor et al. speculated that the most severe damage corresponds to the area of the parafoveal vascular network and that the longer preservation of the avascular area of the fovea may be due to the lack of retinal vessels [3]. According to this theory, HCQ retinopathy begins with the distribution of drugs via the retinal vessels, first affecting the RGCs [21,22].

Clinical studies have shown that the first pathological changes occur in the RGCs of patients using HCQ [23]. However, in our study, the changes in the GCL complex thickness showed no definite correlation with HCQ use. Although this pathophysiological mechanism appears to focus on inner retinal changes in the parafoveal and perifoveal areas of the macula, we observed no significant structural changes in the thickness of the GCL complex. This is because we enrolled early subclinical patients with no VF changes.

This retrospective study is the first to report a concentric five-ring ratio in mfERG to identify the parafoveal region for detecting subclinical HCQ retinopathy. Despite the lack of changes using OCT and VF tests, our results demonstrated that the R1/R3, R1/R4, and R1/R5 ring ratios were equally capable of detecting the electrophysiological changes correlated with the HCQ risk factors, such as the duration of HCQ therapy (*p* = 0.023, 0.048, and 0.045, respectively). Notably, the high proportion of mfERG abnormalities remains difficult to interpret because this indicator of early toxicity may not be detected using other screening tools. Therefore, the AAO guidelines have currently included mfERG among the ancillary tests owing to its lower accessibility [3]. Nonetheless, the coexistence of subtle SS-OCT abnormalities with reduced pericentral mfERG responses, using the ring ratio, can be interpreted as an indicator of early toxicity.

Our results showed that the mfERG test can detect early subtle electrophysiological changes, secondary to retinal cell stress, in patients undergoing HCQ therapy, which may also be an antecedent to the visible photoreceptor and RPE cell damage demonstrable on an OCT image. Consequently, mfERG may be the most sensitive modality for identifying the early signs of HCQ-induced retinal toxicity, even in the absence of clinically detectable retinopathy [24]. Additionally, mfERG is a valuable tool for identifying the correlations between anatomical and functional changes. Thus, improving the test’s efficiency with a simplified interpretation using the R1/R3, R1/R4, and R1/R5 ring ratios will move mfERG one step closer to becoming a routine screening instrument for detecting subclinical HCQ retinopathy.

However, the implications of the early detection of asymptomatic HCQ retinopathy should be debated. Although stopping HCQ can be problematic, especially in patients with SLE, continuing treatment will lead to progressive visual deterioration. Additionally, whether these tests detect a clinically irrelevant loss of photoreceptor cells in a retina in the early stages and whether it would be prudent to reduce the dose of HCQ by 50% in patients with asymptomatic, mild HCQ retinopathy, such as parafoveal damage [5], rather than to stop it remains unknown. Moreover, no point is known beyond which the HCQ toxicity is irreversible. However, in our study, the areas under the ROC curve of the ring ratios (R1/R3, R1/R4, and R1/R5) in the HCQ treatment group between groups 1A and B were 0.730, 0.702, and 0.724, respectively (*p* = 0.004, 0.012, and 0.006). Their cut-off values were 3.84 (R1/R3), 5.60 (R1/R4), and 6.09 (R1/R5), respectively, which indicated a greater significance for the high risk of subclinical HCQ retinopathy. Moreover, this result alone cannot be used to advise discontinuing HCQ medication. However, if we recognize that many asymptomatic or subclinical patients receiving HCQ treatment can be categorized as high-risk, we believe that this will help identify those with the early warning signs. This does not require stopping an HCQ treatment but rather monitoring the patient’s condition carefully because they have more chances of being classified as a high-risk patient. The objective of HCQ-induced retinopathy screening is to recognize the definitive signs of toxicity early to prevent a loss of visual acuity rather than discontinuing valuable drugs at the first borderline abnormality. Thus, our results suggest that various mfERG indicators should be noted in the patients treated with HCQ.

Borrelli et al. reported that the structural changes secondary to HCQ toxicity may occur without clinically obvious retinopathy, which may, in turn, reflect a compromised macular function [24]. They showed that the decrease in inner nuclear layer thickness was related to macular function with the mfERG P1 amplitude in the patients receiving HCQ treatment without retinopathy. However, we found that the ring ratio of the mfERG amplitudes changed in the absence of structural alterations. Moreover, the detectable electrophysiological changes may precede the visible morphological destruction detected via a structural OCT [9,23,25]. Therefore, it has been suggested that mfERG test results represent a better indication of HCQ-induced retinal toxicity.

To the best of our knowledge, this study is valuable for presenting simplified parameters that can be used to identify a high-risk group for subclinical HCQ retinopathy. From the perspective of a rheumatologist, the loss of an important drug, such as HCQ, can cause many restrictions on treatment. Additionally, the implications of the early detection of asymptomatic HCQ retinopathy should be debated, especially as stopping HCQ can be problematic in patients with SLE. Of course, there are no definitive answers to these questions regarding when to discontinue HCQ treatment. Therefore, therapeutic pragmatism with careful monitoring may be the best treatment option for such patients.

Notably, the simplicity of the R1/Rx ratio significantly shortens the test interpretation time, which is needed for improving the testing efficiency and reliability by minimizing the burden. This will help identify the early changes in HCQ-related retinopathy. When these changes occur, the HCQ-treated patients should be monitored closely.

Despite the positive outcomes, this study had some limitations that should be acknowledged. First, owing to the low incidence of HCQ toxicity, our study included a relatively small number of cases from a single tertiary center. Nonetheless, our study included patients without alterations in automated visual field (AVF) tests, such as the HCQ screening modalities, to validate the simplified parameters in mfERG. Second, we used the Humphrey 24-2 AVF instead of the Humphrey 10-2 AVF, which can more sensitively reflect a change in the fovea owing to the retrospective nature of this study. However, wider tests are required for Asian patients, as toxic effects tends to extend beyond the macula area. Further studies should recruit a larger number of patients to validate our results. Third, during the study period, we could not directly measure the sensitivity of the R1/Rx ratio to confirm retinal toxicity using other HCQ screening modalities. Additionally, this study was not sufficiently large to account for confounding factors, such as age, diabetes, or smoking status, which are known to affect mfERG values. Consequently, further longitudinal studies are required to confirm whether the decreased subclinical ring ratios in the mfERG amplitude represent or predict disease progression in patients and whether these patients would benefit from drug discontinuation.

In conclusion, this study focused on the importance of using new ring ratios with mfERG to detect the subclinical alterations of HCQ retinopathy. These mfERG parameters will be valuable for detecting subclinical toxicity in patients with SLE. Future studies with extended longitudinal follow-ups in this cohort may provide additional information. Moreover, while mfERG testing is not widely available due to the complexity of interpreting its results, the use of ring ratios may prove to be a useful biomarker for screening retinal HCQ toxicity.

## Figures and Tables

**Table 1:** Baseline characteristics.

Characteristics	Group 1	Group 1A	Group 1B	Group 2
	All Patients with HCQ Use	Patients with HCQ Use <5 Years	Patients with HCQ Use >5 Years	Controls
Eyes enrolled (patients)	38 (76 eyes)	19 (38 eyes)	19 (38 eyes)	18 (36 eyes)
Age (years)	49.7 ± 14.4	49.4 ± 14.8	49.9 ± 12.4	46.9 ± 16.7
Gender				
Male	1	1	0	1
Female	37	18	19	17
BCVA (logMAR)	0.08 ± 0.07	0.07 ± 0.06	0.09 ± 0.10	0.08 ± 0.06
Duration of HCQ therapy	8.60 ± 6.09	4.05 ± 1.14	13.42 ± 5.48	

HCQ, hydroxychloroquine; BCVA, best-corrected visual acuity.

**Table 2:** The thickness of the macular ganglion cell complex layer in SS-OCT.

Macular Ganglion Cell Complex (μm)	Group 1A	Group 1B	Group 2	*p* Values
	Patients with HCQ Use <5 Years	Patients with HCQ Use >5 Years	Controls	
Superior temporal	69.2 ± 7.16	68.6 ± 6.5	70.1 ± 6.68	0.186
Superior	68.8 ± 4.77	69.1 ± 5.71	70.3 ± 6.44	0.934
Superiornasal	74.1 ± 6.74	74.7 ± 5.68	73.9 ± 7.11	0.828
Inferiortemporal	71.1 ± 5.29	71.6 ± 7.33	71.0 ± 8.18	0.712
Inferior	65.2 ± 4.49	63.6 ± 10.2	67.3 ± 5.8	0.972
Inferior nasal	69.4 ± 6.84	67.5 ± 10.9	67.1 ± 12.8	0.751
Average	69.7 ± 4.76	69.5 ± 6.58	70.3 ± 8.29	0.527

HCQ, hydroxychloroquine; SS-OCT, swept-source optical coherence tomography.

**Table 3:** Amplitudes (P1) in tested multifocal ERG variables in HCQ patients and controls.

ERG Values (nV/deg^2^)	Group 1A	Group 1B	Group 2	*p* Values
	Patients with HCQ Use <5 Years	Patients with HCQ Use >5 Years	Controls	
Ring				
R1	132.0 ± 35.8	125.0 ±39.9	139.0 ± 29.8	0.366
R2	53.0 ± 13.7	52.4 ± 14.0	55.6 ± 16.4	0.853
R3	30.9 ± 8.06	33.1 ± 9.15	30.0 ± 7.36	0.404
R4	22.5 ± 4.38	23.6 ± 5.89	21.9 ± 5.62	0.688
R5	20.4 ± 3.55	21.9 ± 4.97	20.6 ± 6.56	0.253
Quadrants				
1	24.1 ± 4.43	25.9 ± 6.36	25.1 ± 7.54	0.602
2	24.0 ± 5.08	25.4 ± 5.68	24.6 ± 5.33	0.407
3	24.3 ± 5.86	26.1 ± 7.06	25.2 ± 6.9	0.452
4	25.6 ± 5.66	26.8 ± 7.59	22.9 ± 8.01	0.221
Average	24.2 ± 3.59	25.9 ± 5.82	24.2 ± 6.75	0.47

ERG, electroretinography; HCQ, hydroxychloroquine.

**Table 4:** Implicit time (Peak time P1) in tested multifocal ERG variables in HCQ patients and controls.

ERG Values (ms)	Group 1A	Group 1B	Group 2	*p* Values
	Patients with HCQ Use <5 Years	Patients with HCQ Use >5 Years	Controls	
Ring				
R1	40.5 ± 3.12	39.2 ± 2.97	39.1 ± 3.57	0.206
R2	35.4 ± 3.1	35.3 ± 1.9	35.8 ± 3.68	0.811
R3	33.9 ± 1.4	33.9 ± 1.67	35.0 ± 3.63	0.386
R4	36.0 ± 4.4	34.8 ± 1.48	36.4 ± 4.7	0.776
R5	36.3 ± 1.86	36.1 ± 1.53	39.3 ± 6.11	0.488
Quadrants				
1	34.8 ± 1.46	34.9 ± 1.5	36.0 ± 4.76	0.961
2	38.1 ± 4.06	37.1 ± 2.72	39.5 ± 5.76	0.657
3	37.5 ± 4.12	36.8 ± 2.79	37.9 ± 5.12	0.842
4	34.6 ± 1.36	34.7 ± 1.81	36.2 ± 4.77	0.769
Average	36.5 ± 4.42	35.5 ± 1.38	35.5 ± 1.54	0.988

ERG, electroretinography; HCQ, hydroxychloroquine.

**Table 5:** The amplitudes (P1) of the ring ratios in tested multifocal ERG variables in HCQ patients and controls.

ERG Values (nV/deg^2^)	Group 1A	Group 1B	Group 2	*p* Values
	Patients with HCQ Use <5 Years	Patients with HCQ Use >5 Years	Controls	
R1/R1	1	1	1	
R1/R2	2.67 ± 1.01	3.27 ± 3.98	2.69 ± 0.86	0.759
R1/R3	4.58 ± 1.71	4.43 ± 3.41	4.74 ± 1.32	0.018 *
R1/R4	6.06 ± 1.73	5.57 ± 2.03	6.71 ± 2.03	0.029 *
R1/R5	6.61 ± 1.90	6.1 ± 2.57	7.6 ± 3.21	0.029 *

ERG, electroretinography; HCQ, hydroxychloroquine; * *p* value of <0.05 were considered as statistically significant.

## Data Availability

The datasets analyzed during the current study are available from the corresponding authors upon reasonable request.

## References

[1] Willis R., Seif A.M., McGwin G., Martinez-Martinez L.A., González E.B., Dang N., Papalardo E., Liu J., Vilá L.M., Reveille J.D. (2012). Effect of hydroxychloroquine treatment on pro-inflammatory cytokines and disease activity in SLE patients: Data from LUMINA (LXXV), a multiethnic US cohort. Lupus.

[2] Marmor M.F., Kellner U., Lai T.Y., Melles R.B., Mieler W.F. (2016). American Academy of Ophthalmology. Recommendations on Screening for Chloroquine and Hydroxychloroquine Retinopathy (2016 Revision). Ophthalmology.

[3] Marmor M.F., Kellner U., Lai T.Y., Lyons J.S., Mieler W.F., American Academy of Ophthalmology (2011). Revised recommendations on screening for chloroquine and hydroxychloroquine retinopathy. Ophthalmology.

[4] Melles R.B., Marmor M.F. (2014). The risk of toxic retinopathy in patients on long-term hydroxychloroquine therapy. JAMA Ophthalmol..

[5] Marmor M.F., Hu J. (2014). Effect of disease stage on progression of hydroxychloroquine retinopathy. JAMA Ophthalmol..

[6] Kellner S., Weinitz S., Farmand G., Kellner U. (2014). Cystoid macular oedema and epiretinal membrane formation during progression of chloroquine retinopathy after drug cessation. Br. J. Ophthalmol..

[7] Wetterholm D.H., Winter F.C. (1964). Histopathology of chloroquine retinal toxicity. Arch. Ophthalmol..

[8] Bernstein H.N., Ginsberg J. (1964). The pathology of chloroquine retinopathy. Arch. Ophthalmol..

[9] Rosenthal A.R., Kolb H., Bergsma D., Huxsoll D., Hopkins J.L. (1978). Chloroquine retinopathy in the rhesus monkey. Investig. Ophthalmol. Vis. Sci..

[10] Tsang A.C., Ahmadi Pirshahid S., Virgili G., Gottlieb C.C., Hamilton J., Coupland S.G. (2015). Hydroxychloroquine and chloroquine retinopathy: A systematic review evaluating the multifocal electroretinogram as a screening test. Ophthalmology.

[11] Trenkic Božinovic M.S., Stankovic Babic G., Petrovic M., Karadžic J., Šarenac Vulovic T., Trenkic M. (2019). Role of optical coherence tomography in the early detection of macular thinning in rheumatoid arthritis patients with chloroquine retinopathy. J. Res. Med. Sci..

[12] Turgut B., Turkcuoglu P., Serdar Koca S., Aydemir O. (2009). Detection of the regression on hydroxychloroquine retinopathy in optical coherence tomography. Clin. Rheumatol..

[13] Aydın Kurna S., Kanar H.S., Garlı M., Çakır N. (2022). Evaluation of the role of spectral-domain optical coherence tomography in the early detection of macular and ganglion cell complex thickness changes in patients with rheumatologic diseases taking hydroxychloroquine. Photodiagnosis Photodyn. Ther..

[14] Hoffmann M.B., Bach M., Kondo M., Li S., Walker S., Holopigian K., Viswanathan S., Robson A.G. (2021). ISCEV standard for clinical multifocal electroretinography (mfERG) (2021 update). Doc. Ophthalmol..

[15] Ozawa H., Ueno S., Ohno-Tanaka A., Sakai T., Hashiguchi M., Shimizu M., Fujinami K., Ahn S.J., Kondo M., Browning D.J. (2021). Ocular findings in Japanese patients with hydroxychloroquine retinopathy developing within 3 years of treatment. Jpn. J. Ophthalmol..

[16] Kim J., Jeong H.C., Kwon H.Y., Kim Y.H., Ahn S.J. (2024). Demographic and clinical characteristics associated with screening practices for hydroxychloroquine retinopathy. Sci. Rep..

[17] Payne J.F., Hubbard G.B., Aaberg T.M., Yan J. (2011). Clinical characteristics of hydroxychloroquine retinopathy. Br. J. Ophthalmol..

[18] Michaelides M., Stover N.B., Francis P.J., Weleber R.G. (2011). Retinal toxicity associated with hydroxychloroquine and chloroquine: Risk factors, screening, and progression despite cessation of therapy. Arch. Ophthalmol..

[19] Kellner U., Kellner S., Weinitz S. (2008). Chloroquine retinopathy: Lipofuscin- and melanin-related fundus autofluorescence, optical coherence tomography and multifocal electroretinography. Doc. Ophthalmol..

[20] Kellner S., Weinitz S., Kellner U. (2009). Spectral domain optical coherence tomography detects early stages of chloroquine retinopathy similar to multifocal electroretinography, fundus autofluorescence and near-infrared autofluorescence. Br. J. Ophthalmol..

[21] Mahon G.J., Anderson H.R., Gardiner T.A., McFarlane S., Archer D.B., Stitt A.W. (2004). Chloroquine causes lysosomal dysfunction in neural retina and RPE: Implications for retinopathy. Curr. Eye Res..

[22] Borrelli E., Battista M., Cascavilla M.L., Viganò C., Borghesan F., Nicolini N., Clemente L., Sacconi R., Barresi C., Marchese A. (2021). Impact of structural changes on multifocal electroretinography in patients with use of hydroxychloroquine. Investig. Ophthalmol. Vis. Sci..

[23] Tsang A.C., Ahmadi S., Hamilton J., Gao J., Virgili G., Coupland S.G., Gottlieb C.C. (2019). The diagnostic utility of multifocal electroretinography in detecting chloroquine and hydroxychloroquine retinal toxicity. Am. J. Ophthalmol..

[24] Adam M.K., Covert D.J., Stepien K.E., Han D.P. (2012). Quantitative assessment of the 103-hexagon multifocal electroretinogram in detection of hydroxychloroquine retinal toxicity. Br. J. Ophthalmol..

[25] Browning D.J., Lee C. (2014). Relative sensitivity and specificity of 10-2 visual fields, multifocal electroretinography, and spectral domain optical coherence tomography in detecting hydroxychloroquine and chloroquine retinopathy. Clin. Ophthalmol..

